# Exercise leads to unfavourable cardiac remodelling and enhanced metabolic homeostasis in obese mice with cardiac and skeletal muscle autophagy deficiency

**DOI:** 10.1038/s41598-017-08480-2

**Published:** 2017-08-11

**Authors:** Zhen Yan, Ana Kronemberger, Jay Blomme, Jarrod A. Call, Hannah M. Caster, Renata O. Pereira, Henan Zhao, Vitor U. de Melo, Rhianna C. Laker, Mei Zhang, Vitor A. Lira

**Affiliations:** 1 Department of Health & Human Physiology, Obesity Research and Educational Initiative, Fraternal Order of Eagles Diabetes Research Center, University of Iowa, Iowa City, IA United States; 2 Departments of Medicine, Pharmacology, Molecular Physiology & Biological Physics, Robert M. Berne Cardiovascular Research Center, University of Virginia School of Medicine, Charlottesville, VA United States; 3 Department of Kinesiology & Regenerative Bioscience Center, University of Georgia, Athens, GA United States; 4 Department of Internal Medicine, Carver College of Medicine, University of Iowa, Iowa City, IA United States; 5 Department of Physiology, Federal University of Sergipe, São Cristóvão, SE Brazil

## Abstract

Autophagy is stimulated by exercise in several tissues; yet the role of skeletal and cardiac muscle-specific autophagy on the benefits of exercise training remains incompletely understood. Here, we determined the metabolic impact of exercise training in obese mice with cardiac and skeletal muscle disruption of the Autophagy related 7 gene (Atg7^h&mKO^). Muscle autophagy deficiency did not affect glucose clearance and exercise capacity in lean adult mice. High-fat diet in sedentary mice led to endoplasmic reticulum stress and aberrant mitochondrial protein expression in autophagy-deficient skeletal and cardiac muscles. Endurance exercise training partially reversed these abnormalities in skeletal muscle, but aggravated those in the heart also causing cardiac fibrosis, foetal gene reprogramming, and impaired mitochondrial biogenesis. Interestingly, exercise-trained Atg7^h&mKO^ mice were better protected against obesity and insulin resistance with increased circulating fibroblast growth factor 21 (FGF21), elevated *Fgf21* mRNA and protein solely in the heart, and upregulation of FGF21-target genes involved in thermogenesis and fatty acid oxidation in brown fat. These results indicate that autophagy is essential for the protective effects of exercise in the heart. However, the atypical remodelling elicited by exercise in the autophagy deficient cardiac muscle enhances whole-body metabolism, at least partially, via a heart-brown fat cross-talk involving FGF21.

## Introduction

The benefits of an active lifestyle to human health are well known. Exercise positively affects metabolism and function of a number of tissues improving whole-body metabolic homeostasis and reducing overall disease risk^1, 2^. However, the underlying cellular and molecular mechanisms are complex and incompletely understood. Interestingly, mild calorie restriction shares certain metabolic benefits provided by exercise, such as improved insulin sensitivity, reduced overall oxidative stress, and enhanced tissue and cellular function. Those are in clear contrast with the whole-body and cellular metabolic derangements observed with chronic over nutrition and aging^3, 4^, which are major contributors to the development of obesity and type II diabetes. Therefore, it is not surprising that exercise and calorie restriction remain the primary line of preventive measures, as well as important therapeutics for chronic metabolic diseases^5^.

Macroautophagy, hereafter referred to as autophagy, is a catabolic process conserved from yeast to mammalian cells that is required for removal of aggregate proteins and dysfunctional organelles in a lysosomal-dependent manner^6, 7^. As such, autophagy is instrumental for both protein and organelle quality control. Increased levels of autophagy have been implicated as a central life-extending mechanism for calorie restriction and calorie restriction mimetics in different species^8^. Accordingly, endurance exercise stimulates autophagy in several tissues including skeletal muscle and heart^9, 10^.

Studies with systemic impairments in autophagy regulation indicate that autophagy is required for exercise-induced mitochondrial biogenesis and angiogenesis in skeletal muscle of lean mice^11^, and that it is necessary for exercise-mediated preservation of insulin sensitivity in obese mice^10^. Although these studies clearly highlight the importance of overall autophagy for the metabolic adaptations to exercise, the potential role of cardiac and skeletal muscle-specific autophagy in those adaptations remains to be determined.

Here, we investigated the exercise protection against the metabolic insults of high-fat diet (HFD) feeding in mice with selective cardiac and skeletal muscle autophagy related 7 gene (*Atg7*) deficiency. Since ATG7 is required for the essential autophagy step of autophagosome formation^12^, we hypothesized that the insulin-sensitizing and cardioprotective effects of exercise would be absent in these muscle autophagy-deficient animals. Our results reveal that exercise training indeed leads to unfavourable remodelling in cardiac muscle, but not in skeletal muscle with impaired autophagy. Additionally, our findings reveal that the whole-body metabolic benefits of exercise are enhanced in obese mice with cardiac and skeletal muscle autophagy deficiency, at least in part, because of a heart-brown adipose tissue cross-talk involving fibroblast growth factor 21 (FGF21).

## Results

### Impaired autophagy in skeletal and cardiac muscles does not affect glucose clearance and exercise capacity in adult mice

**Figure 1:**
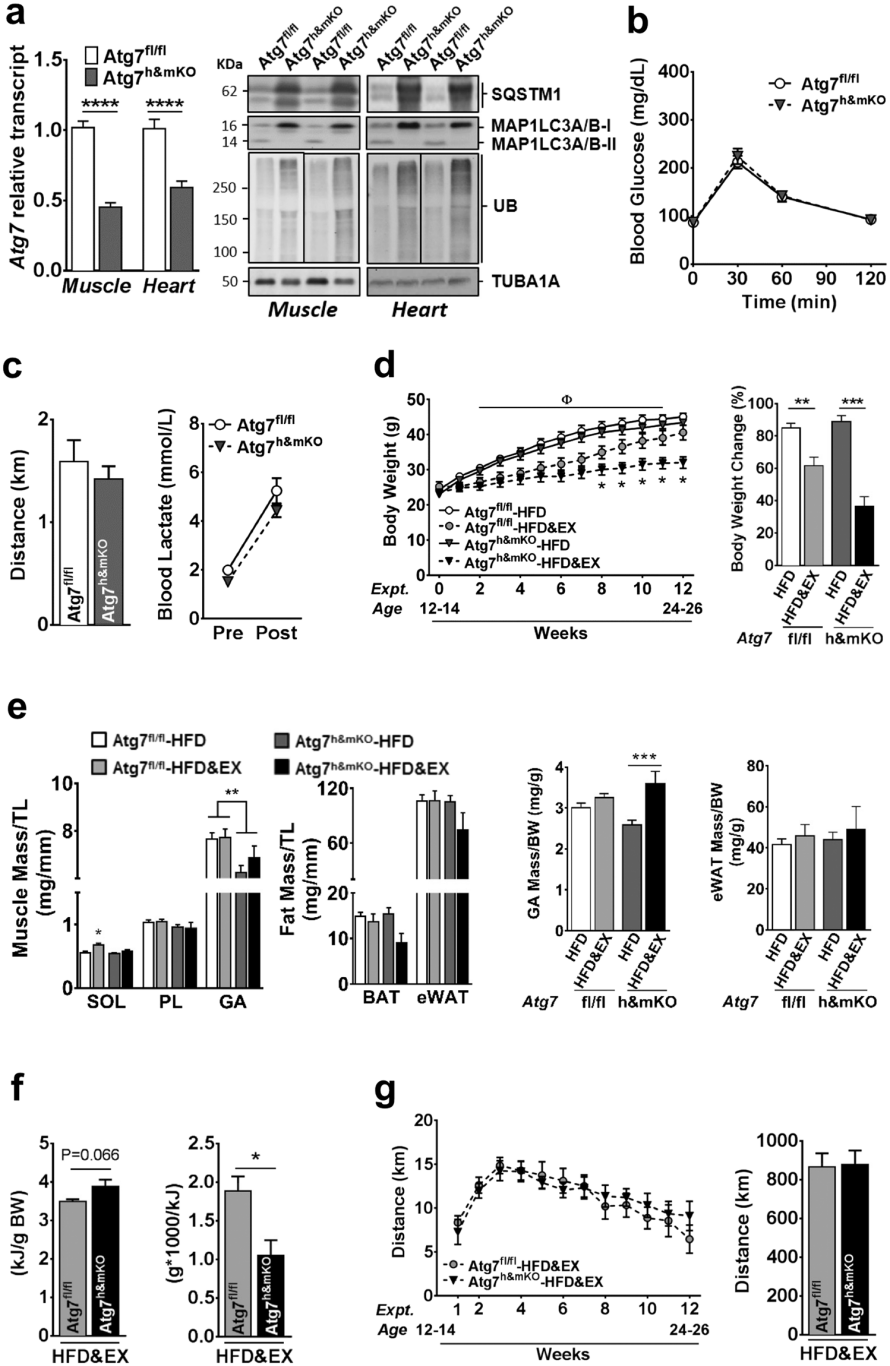
Diet-induced obesity in exercise-trained Atg7^h&mKO^ mice. (**a**) *Atg7* mRNA and immunoblot representative images of the mean value for select autophagy and ubiquitinated proteins (UB) in muscle and heart (n = 11–15). (**b**) GTT (n = 14). (**c**) Distance run and blood lactate before (Pre) and after (Post) the endurance exercise capacity test (n = 11). (**d**) Body weight quantification and percentage change during the intervention (n = 8–11). (**e**) Muscle (Soleus-SOL, Plantaris-PL, Gastrocnemius-GA) and adipose (BAT and eWAT) masses normalized to tibia length (TL) and body weight (BW) (n = 6–11). (**f**) Average daily caloric intake, and feed efficiency (n = 6). (**g**) Weekly average of daily voluntary running activity, and total running distance during the intervention (n = 6). *P < 0.05, **P < 0.01, ***P < 0.001 ****P < 0.0001 in comparison to all other groups, unless otherwise represented; ^**Φ**^ *P* < 0.05 both Atg7^fl/fl^-HFD&EX and Atg7^h&mKO^-HFD&EX in comparison to respective HFD only, sedentary groups.

Atg7^h&mKO^ mice presented an approximate 55% and 40% reduction of *Atg7* mRNA expression in skeletal muscle and in the heart, respectively (Fig. 1a). Basal autophagy was substantially reduced in both tissues as evidenced by a large sequestosome 1 (SQSTM1, a.k.a. p62) accumulation, non-detectable conversion of microtubule-associated protein 1 light chain 3 alpha and beta-I (MAP1LC3A/B-I, a.k.a. LC3A/B-I) into MAP1LC3A/B-II (a.k.a. LC3A/B-II), and accumulation of ubiquitinated proteins (Fig. 1a, Supplementary Fig. S1a). Despite the potential functional and metabolic impacts of reduced autophagy^13^, adult Atg7^h&mKO^ animals (12–14 weeks of age) were ~5% lighter than control littermates (Atg7^fl/fl^; Supplementary Fig. S1b), but presented normal glucose handling and endurance exercise capacity (Fig. 1b,c).

### Exercise-mediated protection against diet-induced obesity and insulin resistance is potentiated in Atg7^h&mKO^

We next evaluated whether the benefits of endurance exercise counteracted the detrimental effects of diet-induced obesity and insulin resistance in Atg7^h&mKO^. All mice gained weight during the intervention, with sedentary control and Atg7^h&mKO^ almost doubling their body weight. Exercise training prevented weight gain more effectively in Atg7^h&mKO^ (Fig. 1d). Atg7^h&mKO^ tended to have slightly lower muscle masses than control mice, but exercise training increased muscle mass to body weight ratio in Atg7^h&mKO^, indicating a preferential protection against gains in fat mass (Fig. 1e). This obesity-resistant phenotype of exercise-trained Atg7^h&mKO^ was not caused by either increased running activity or reduced food intake when compared to exercise-trained control mice (Fig. 1f,g).

**Figure 2:**
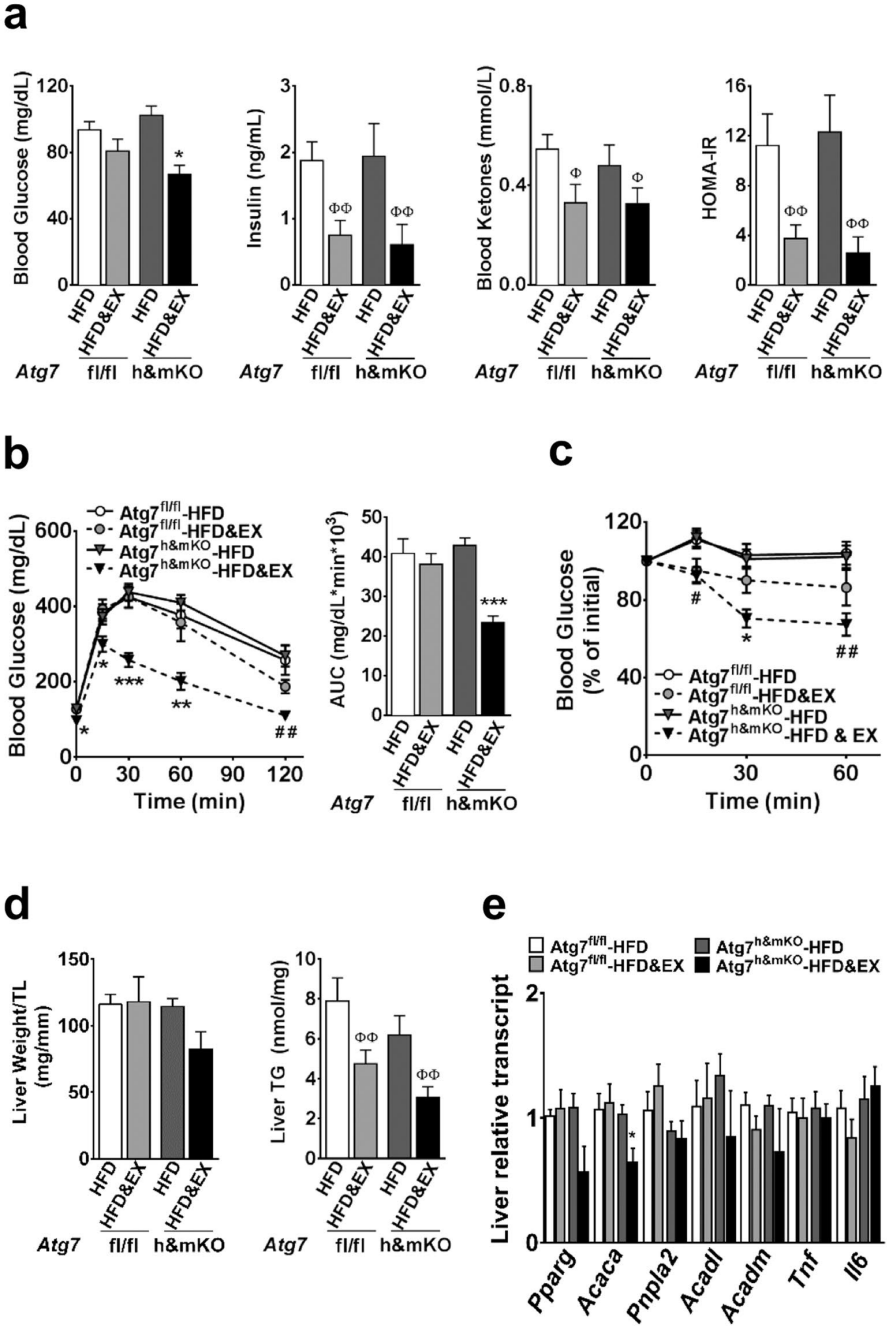
Improved whole-body glucose homeostasis in exercise-trained Atg7^h&mKO^ mice. (**a**) Overnight fasting blood levels of glucose, insulin, and ketones, and resulting HOMA-IR (n = 5–11). (**b**) GTT and respective area under the curve (AUC) (n = 8–11). (**c**) ITT (n = 8–11). (**d**) Liver mass normalized to tibia length (TL), and liver triglyceride (TG) content (n = 5–11). (**e**) mRNA of hepatic genes involved in lipid homeostasis and inflammation (n = 5–9). *P < 0.05, **P < 0.01 and ***P < 0.001 in comparison to all other groups; ^**#**^ *P* < 0.05, ^**##**^ *P* < 0.01 in comparison to Atg7^fl/fl^-HFD and Atg7^fl/fl^-HFD&EX groups; and ^**Φ**^ *P* < 0.05, ^**ΦΦ**^ *P* < 0.01 in comparison to respective HFD only, sedentary group.

Exercise lowered fasting blood glucose, insulin and ketones in both control and Atg7^h&mKO^ mice (Fig. 2a). However, exercise-trained Atg7^h&mKO^ mice had improved glucose clearance capacity and insulin sensitivity when further challenged during glucose and insulin tolerance tests (Fig. 2b,c). Exercise training diminished hepatic triglyceride accumulation in both control and Atg7^h&mKO^ mice, but these effects tended to be more prominent in Atg7^h&mKO^ (Fig. 2d). Hepatic gene expression revealed that this phenomenon was not associated with increased lipolysis or β-oxidation, but rather reduced *de novo* lipid synthesis indicated by low expression of acetyl-coenzyme A carboxylase alpha (*Acaca*) and a trend for reduced peroxisome proliferator activated receptor gamma (*Pparg*) in exercise-trained Atg7^h&mKO^ (Fig. 2e).

### Exercise causes a distinctive adipose gene profile associated with increased circulating FGF21 in Atg7^h&mKO^ mice

**Figure 3:**
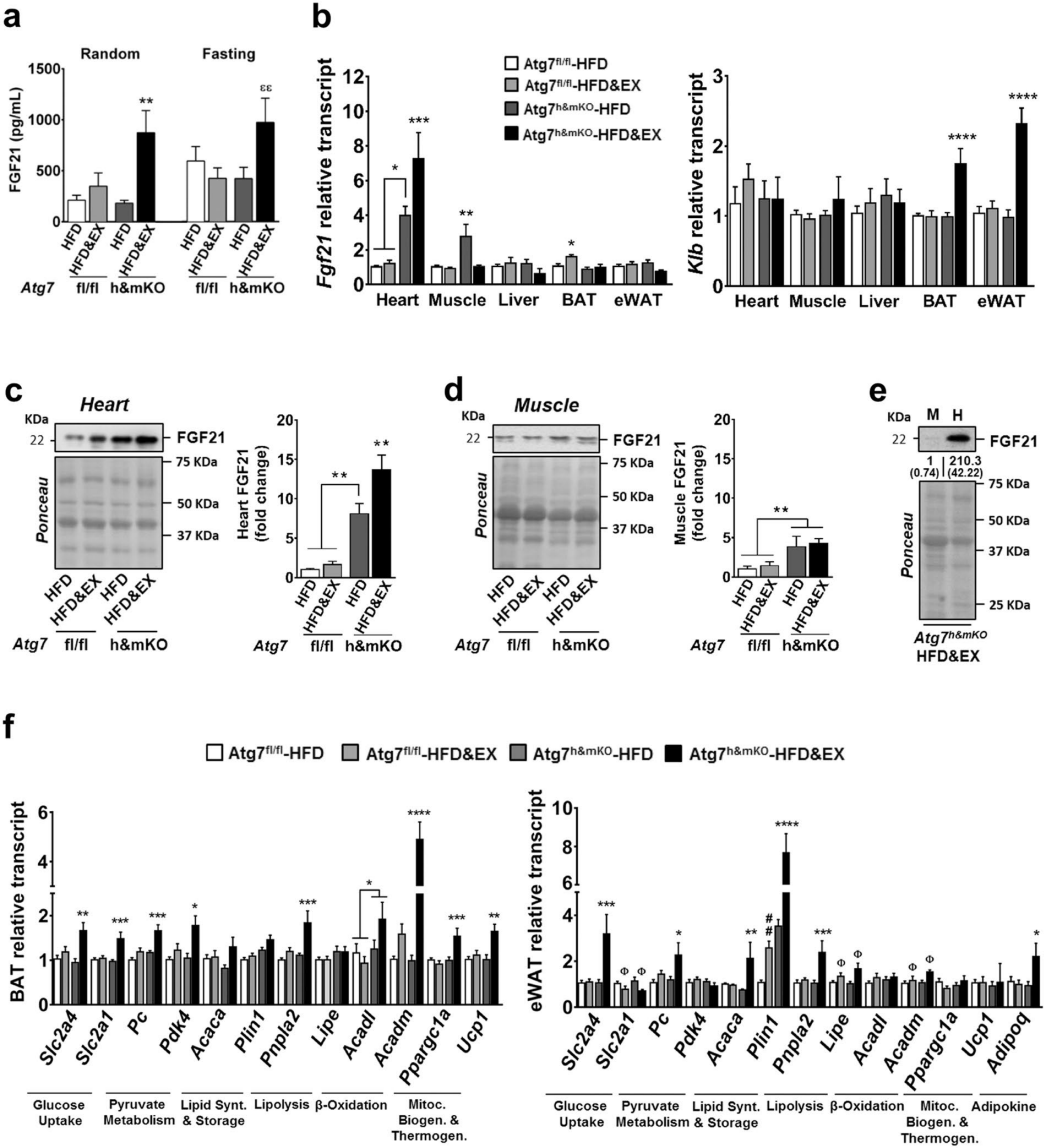
Elevated circulating FGF21 and associated BAT and eWAT metabolic gene profile in exercise-trained Atg7^h&mKO^ mice. (**a**) Circulating FGF21 under random feeding and overnight fasting conditions (n = 5–7). (**b**) tissue-specific mRNA analysis of *Fgf21* and *Klb* (n = 5–10). (**c**) Immunoblot representative images of the mean value and related quantification of cardiac FGF21 (n = 5). (**d**) Immunoblot representative images of the mean value and related quantification of gastrocnemius skeletal muscle FGF21 (n = 5). (**e**) Immunoblot representative images of the mean value and related quantification of muscle (M) vs. heart (H) FGF21 levels in exercise-trained Atg7 Atg7^h&mKO^ mice (n = 3). (**f**) mRNA analysis of select metabolic genes in BAT and eWAT (n = 6–10). Full-length blots are presented in Supplementary Fig. S4. *P < 0.05, **P < 0.01, ***P < 0.001, ****P < 0.0001 in comparison to all other groups, unless otherwise represented; ^**##**^ *P* < 0.01 in comparison to Atg7^fl/fl^-HFD; and ^Φ^ *P* < 0.05 denotes a significant effect of exercise in both Atg7^fl/fl^ and Atg7^h&mKO^; and ^**εε**^ *P* < 0.01 in comparison to Atg7^h&mKO^-HFD and Atg7^fl/fl^-HFD&EX groups.

The fact that exercise-trained Atg7^h&mKO^ presented comparable food intake and running activity, but were protected against obesity and insulin resistance when compared to control mice, suggested these mice may have an accelerated metabolic rate. Because deficient autophagy in muscle or liver can lead to increased circulating FGF21 promoting thermogenic phenotypes in adipose tissues^14, 15^, we next assessed serum FGF21 levels in control and Atg7^h&mKO^. FGF21 was elevated by ~4.5-fold in random feeding and by ~2-fold in fasted conditions in exercise-trained Atg7^h&mKO^ mice (Fig. 3a). To determine the source of increased circulating FGF21, we examined *Fgf21* in several tissues. *Fgf21* was unchanged in adipose tissues and in the liver, and was elevated in skeletal muscle only in sedentary Atg7^h&mKO^ mice. Curiously, *Fgf21* was increased in the heart of sedentary Atg7^h&mKO^, but further enhanced by exercise training (Fig. 3b, left). Muscle FGF21 presented a slight different pattern from muscle *Fgf21*, as it was increased in both sedentary and exercise-trained Atg7^h&mKO^ mice (Fig. 3d). However, cardiac FGF21 closely resembled cardiac *Fgf21*, being dramatically increased in exercise-trained Atg7^h&mKO^ mice relative to all other groups (Fig. 3c), and was >200-fold higher than in skeletal muscle (Fig. 3e). Altogether, these results point to the heart as the primary source of increased circulating FGF21 in exercise-trained Atg7^h&mKO^ mice. Adipose tissue klotho beta (*Klb*, a.k.a. *β-Klotho*), the specific co-receptor for FGF21 that is known to be reduced in diet-induced obesity^16^, was also present at higher levels in exercise-trained Atg7^h&mKO^ mice (Fig. 3b, right). We then examined the metabolic gene expression profile of the interscapular brown adipose tissue, and of the visceral, epididymal white adipose tissue (BAT and eWAT, respectively).

Exercise training elevated the expression of Pparg coactivator 1 alpha (*Ppargc1a*) and uncoupling protein 1 (*Ucp1*) in BAT of Atg7^h&mKO^ mice, indicative of enhanced thermogenesis. Further changes in BAT gene expression included high levels of both solute carrier family 2 members 1 and 4 (*Slc2a1*, a.k.a. *Glut1*, and *Slc2a4*, a.k.a. *Glut4*, respectively), denoting increased glucose uptake capacity under both basal and insulin-stimulated conditions. These were accompanied by elevated expression of both pyruvate carboxylase (*Pc*) and pyruvate dehydrogenase kinase 4 (*Pdk4*) (Fig. 3c, left), suggesting a stimulated conversion of pyruvate to oxaloacetate and a parallel inhibition of pyruvate conversion to acetyl-CoA^17^. Collectively, these findings point to a preferential use of pyruvate as an anaplerotic substrate in the Krebs cycle to sustain fatty acid oxidation. Accordingly, exercise-trained Atg7^h&mKO^ displayed a substantial elevation in the expression of the β-oxidation genes acyl-CoA dehydrogenases of medium and long chain fatty acids (*Acadm* and *Acadl*, respectively), as well as upregulation of the fatty acid mobilization gene patatin like phospholipase domain containing 2 (*Pnpla2*, a.k.a. *Atgl*) in BAT (Fig. 3f, left). Overall, these are consistent with enhanced FGF21 signalling leading to elevated fatty acid oxidation and thermogenesis in BAT of exercise-trained Atg7^h&mKO^.

However, exercise training did not alter *Ppargc1a* or *Ucp1* in eWAT of Atg7^h&mKO^ mice. Furthermore, exercise training reduced the expression of *Slc2a1* in eWAT of both control and Atg7^h&mKO^ mice, but elevated the expression of *Slc2a4* in the same depot. This was accompanied by upregulation of *Pc* without changes in *Pdk4* (Fig. 3c, right), suggesting that epididymal white adipocytes of exercise-trained Atg7^h&mKO^ were prioritizing the conversion of pyruvate to both oxaloacetate and acetyl-CoA, which are substrates for citrate synthesis. Such increased potential for citrate synthesis was likely geared towards *de novo* lipid synthesis rather than oxidation, as evidenced by increased expression of *Acaca* and substantial induction of perilipin 1 (*Plin1*), required for triglyceride storage. The adipokine adiponectin (*Adipoq*) was elevated by ~2-fold in eWAT of exercise-trained Atg7^h&mKO^ as well. These results are in line with an elevated insulin-dependent glucose uptake and lipid storage capacity in eWAT of exercise-trained Atg7^h&mKO^ mice, but do not indicate that FGF21 was stimulating thermogenesis and/or substrate oxidation in this tissue.

Of note, elevated adipose tumor necrosis factor (TNF) is a feature of the obesity-associated pro-inflammatory milieu that is known to repress the expression of *Slc2a4* ^18^, *Plin1* ^19^ and *Klb* ^20^; all of which were enhanced in exercise-trained Atg7^h&mKO^ mice. Adipose *Pparg* ^21^, however, stimulates the expression of these genes and is also repressed by adipose *Tnf*. Here, we observed a parallel reduction of *Tnf* with elevations of *Pparg* in both eWAT and BAT of exercise-trained Atg7^h&mKO^ mice (Supplementary Fig. S2a). Collectively, these findings suggest that the increased circulating FGF21 also helped mitigate inflammation in adipose tissues, which might underlie the preserved eWAT glucose uptake and lipid storage capacities in exercise-trained Atg7^h&mKO^ mice.

### Exercise-trained Atg7^h&mKO^ mice had preserved insulin signalling and restored mitochondrial biogenesis in skeletal muscle

**Figure 4:**
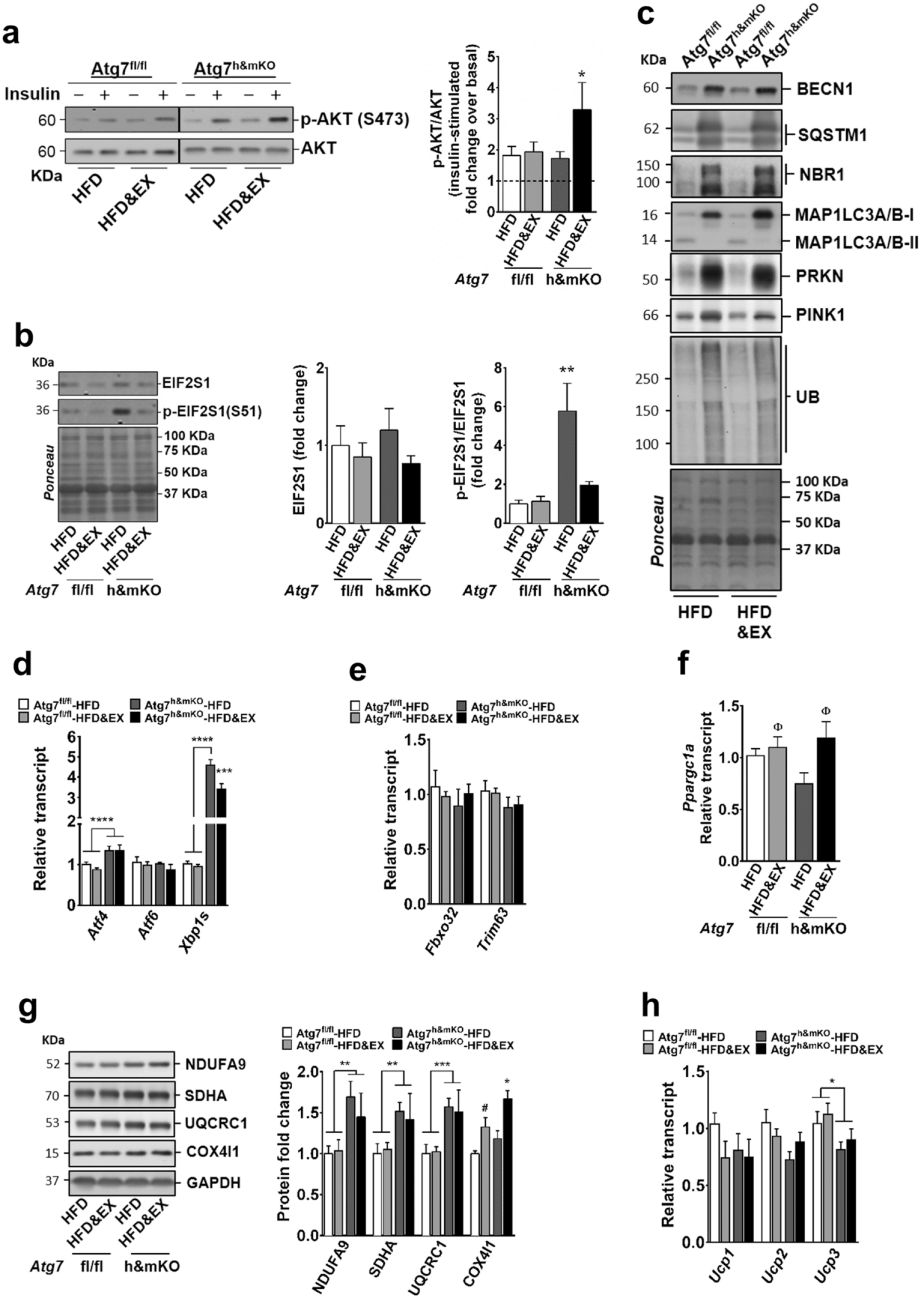
Insulin signalling, ER stress and mitochondrial biogenesis are favourably modulated by exercise training in skeletal muscle of Atg7^h&mKO^ mice. (**a**) Immunoblot representative images of the mean value (dividing lines indicate blots from different gels) and related quantification of phosphorylated AKT(S473) normalized to total AKT under basal and insulin-stimulated conditions (dashed line represents basal AKT phosphorylation) (n = 6–11). (**b**) Immunoblot representative images of the mean value and related quantification of total EIF2S1 normalized to ponceau staining, and phosphorylated EIF2S1(S51) normalized to total EIF2S1 (n = 5–6). (**c**) Immunoblot representative images of the mean value for select autophagy, mitophagy and ubiquitinated proteins (n = 4). (**d**) mRNA analysis of ER stress effector genes (n = 5–9). (**e**) mRNA analysis of E3 ubiquitin ligase genes (n = 5–9). (**f**) mRNA analysis of *Ppargc1a* (n = 5–9). (**g**) Immunoblot representative images of the mean value and quantification of mitochondrial proteins of complexes 1–4 of the Electron Transport Chain normalized to glyceraldehyde 3-phosphate dehydrogenase (GAPDH; n = 5–10). (**h**) mRNA analysis of *Ucp1*, *Ucp2* and *Ucp3* (n = 5–9). Full-length blots are presented in Supplementary Fig. S5. *P < 0.05, **P < 0.01, ***P < 0.001, ****P < 0.0001 in comparison to all other groups unless otherwise represented; ^Φ^ *P* < 0.05 in comparison to respective HFD only, sedentary group; and ^#^ *P* < 0.05 in comparison to Atf7^fl/fl^-HFD.

Exercise training enhanced insulin-stimulated phosphorylation of AKT (S473) in skeletal muscle of Atg7^h&mKO^ mice indicating preserved muscle insulin sensitivity compared to control mice on HFD (Fig. 4a, Supplementary Fig S2b). Both HFD and deficient autophagy have been shown to cause endoplasmic reticulum (ER) stress^13, 14, 22^. Examination of key molecular effectors of the ER stress response in sedentary muscles of Atg7^h&mKO^ revealed increased levels of phosphorylated eukaryotic translation initiation factor 2 subunit alpha (EIF2S1, a.k.a. eIF2α, at S51), as well as induction of activating transcription factor 4 (*Atf4*), and spliced X-box binding protein 1 (*Xbp1s*). Exercise training restored EIF2S1 phosphorylation levels of Atg7^h&mKO^ to that of control mice and reduced *Xbp1s*, consistent with a partial reversal of ER stress (Fig. 4b,d). Such reduction of ER stress was not caused by an improvement in the canonical autophagy pathway because exercise training did not enhance MAP1LC3A/B-I to MAP1LC3A/B-II conversion or reduced the accumulation of the adaptor proteins SQSTM1 and neighbour of BRCA1 gene 1 (NBR1) in skeletal muscle (Fig. 4c). We next tested whether exercise may have generated a compensatory stimulation of the alternative autophagy pathway and/or the ubiquitin-proteasome system. In that sense, beclin 1 (BECN1), involved in both canonical and alternative autophagy^23^, was elevated to a similar degree in sedentary and exercise-trained Atg7^h&mKO^ negating a preferential stimulation of alternative autophagy by exercise. Likewise, accumulation of ubiquitinated proteins, which can be caused by impairments in autophagy and/or ubiquitin-proteasome system, did not change with exercise training in Atg7^h&mKO^ (Fig. 4c). Analysis of the transcriptionally regulated muscle-specific E3 ubiquitin ligases F-box protein 32 (*Fbxo32*, a.k.a. *Atrogin1*) and tripartite motif containing 63 (*Trim63*, a.k.a. *Murf1*) also failed to reveal a selective stimulation of the ubiquitin-proteasome system by exercise training in Atg7^h&mKO^ (Fig. 4e). Together, these results do not support a compensatory upregulation of overall protein degradation as a central mechanism underlying the partial ER stress reversal by exercise training in skeletal muscle of Atg7^h&mKO^ mice. Because mitochondria are directly targeted by autophagy, and mitochondrial dysfunction can lead to ER stress, exercise may have reduced ER stress by acting upon mitochondrial protein homeostasis. PINK1 and parkin (PRKN), instrumental proteins for degradation of mitochondria by autophagy (i.e. mitophagy) that are also involved in the select degradation of certain mitochondrial proteins by the ubiquitin-proteasome system^24, 25^, were increased in both sedentary and exercise-trained Atg7^h&mKO^ (Fig. 4c, Supplementary Fig. S2c), opposing a preferential stimulation of mitochondrial protein degradation by exercise. Nevertheless, sedentary Atg7^h&mKO^ mice had impaired mitochondrial biogenesis and an aberrant mitochondrial protein expression; features that were either completely or partially reversed by exercise training. This is evidenced by a trend towards reduced *Ppargc1a*, which regulates mitochondrial biogenesis and its own expression^26^, and a parallel increase of components of the electron transport chain complexes 1, 2 and 3 (NADH:ubiquinone oxidoreductase subunit A9 - NDUFA9, Succinate dehydrogenase complex flavoprotein subunit A – SDHA, and Ubiquinol-Cytochrome C Reductase Core Protein 1 - UQCRC1, respectively) in sedentary Atg7^h&mKO^ versus control mice. Exercise training restored *Ppargc1a* and increased cytochrome c oxidase subunit 4 isoform 1 (COX4I1, a component of the electron transport chain complex 4) in Atg7^h&mKO^ mice and in the controls (Fig. 4f,g). Altogether, these findings suggest that improved mitochondrial protein homeostasis supported by mitochondrial biogenesis may underlie the reduced ER stress seen with exercise training in skeletal muscle with impaired autophagy.

We next investigated if skeletal muscle of exercise-trained Atg7^h&mKO^ mice was contributing to the observed resistance to obesity. To test this possibility, we assessed the expression of *Ucp* genes and the expression of putative myokines known to modulate adipose tissue metabolism. First, *Ucp1* and *Ucp2* were not different across groups, whereas *Ucp3* was reduced in both sedentary and exercise-trained Atg7^h&mKO^ mice (Fig. 4h). Second, fibronectin type III domain containing 5 (*Fndc5*)^27^, but not interleukin 15 (*Il15*)^28^ and meteorin, glial cell differentiation regulator-like (*Metrnl*)^29^, was moderately increased by exercise training. However, this was seen in both control and Atg7^h&mKO^ mice, which is not consistent with a preferential contribution of *Fndc5* to the more pronounced obesity-resistant phenotype caused by exercise training in autophagy deficient mice (Supplementary Fig. S2c). These results suggest that skeletal muscle directly contributed to the insulin-sensitive, but not to the obesity-resistant, phenotype of exercise-trained Atg7^h&mKO^ mice.

### Exercise leads to unfavourable cardiac remodelling in Atg7^h&mKO^ mice

**Figure 5:**
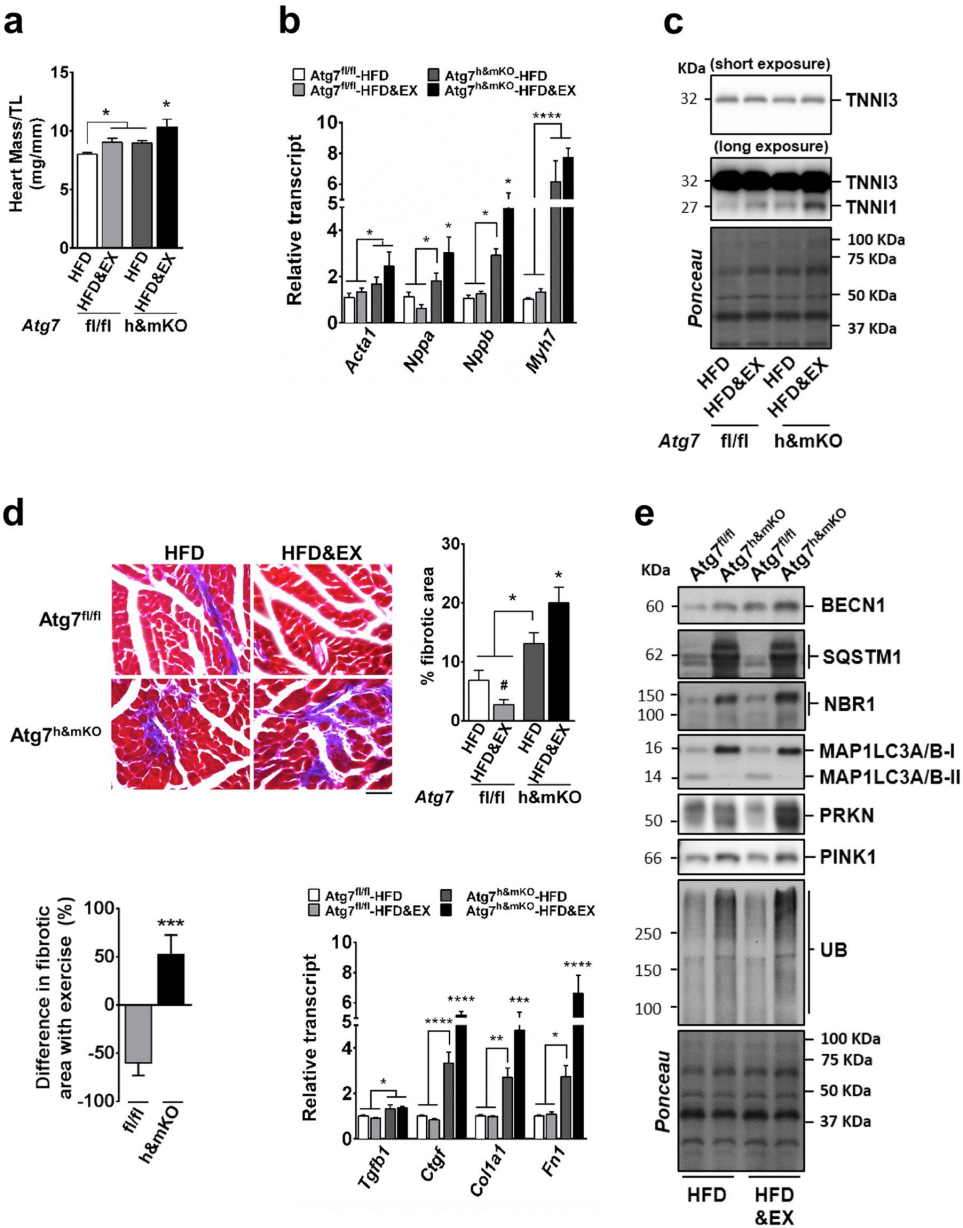
Exercise causes unfavourable cardiac remodelling in Atg7^h&mKO^ mice. (**a**) Heart mass normalized to tibia length (TL) (n = 6–11). (**b**) mRNA analysis of foetal genes (n = 5–8). (**c**) Immunoblot representative images of the mean value for cardiac troponin I3 (TNNI3) and skeletal muscle TNNI1 (n = 4). (**d**) Representative micrographs of Masson’s trichrome stain showing fibrosis in left ventricle sections (Scale bar = 100 μm), and quantification of fibrotic area represented as a percentage of total area, quantification of the difference in fibrotic area with exercise training for each genotype, and mRNA analysis of genes involved in fibrosis (n = 5–8). (**e**) Immunoblot representative images of the mean value for select autophagy, mitophagy and ubiquitinated proteins (n = 4). Full-length blots are presented in Supplementary Fig. S6. *P < 0.05, **P < 0.01, ***P < 0.001 and ****P < 0.001 in comparison to all other groups, unless otherwise represented; ^**#**^ *P* < 0.05 in comparison to Atf7^fl/fl^-HFD.

When compared with control mice, sedentary Atg7^h&mKO^ mice had larger hearts with elevated expression of the foetal program genes skeletal muscle actin alpha 1 (*Acta1*), natriuretic peptides A and B (*Nppa*, a.k.a. *Anp*, and *Nppb*, a.k.a. *Bnp*), and myosin heavy chain 7 (*Myh7*), which are indicative of pathological hypertrophy. These changes, however, did not affect circulating levels of either NPPA or NPPB (Supplementary Fig. S3a), which could have caused beneficial metabolic adaptations in adipose tissues^30^. Exercise training increased heart mass in both control and Atg7^h&mKO^ mice, but increased the expression of foetal genes only in Atg7^h&mKO^ mice, suggesting an exacerbation of pathological hypertrophy (Fig. 5a,b). Elevated slow skeletal troponin I (TNNI1) expression in exercise-trained Atg7^h&mKO^ mice hearts provides further biochemical evidence for an activated foetal program^31, 32^ indicating maladaptive cardiac events in these animals (Fig. 5c). Accordingly, exercise training reduced by ~60% diet-induced cardiac fibrosis in control mice, whereas it increased fibrosis by ~50% and elevated expression of genes associated with a fibrotic phenotype in Atg7^h&mKO^ mice (Fig. 5d).

Next, we examined mechanisms potentially responsible for these maladaptive events caused by exercise training in Atg7^h&mKO^ mice. Similar to skeletal muscle, cardiac BECN1 expression was increased in both sedentary and exercise-trained Atg7^h&mKO^ mice when compared to respective controls, without obvious effects on autophagy (both canonical and alternative) or the ubiquitin proteasome system, as indicated by impaired MAP1LC3A/B-I to MAP1LC3A/B-II conversion and accumulation of SQSTM1, NBR1 and ubiquitinated proteins. Also similar to skeletal muscle, cardiac PRKN and PINK1 were increased in both sedentary and exercise-trained Atg7^h&mKO^ mice (Fig. 5e). Together, these results suggest that cardiac autophagy deficiency led to impairments in overall protein degradation, as well as stimulation of potential compensatory mechanisms that were not differently affected by exercise.

**Figure 6:**
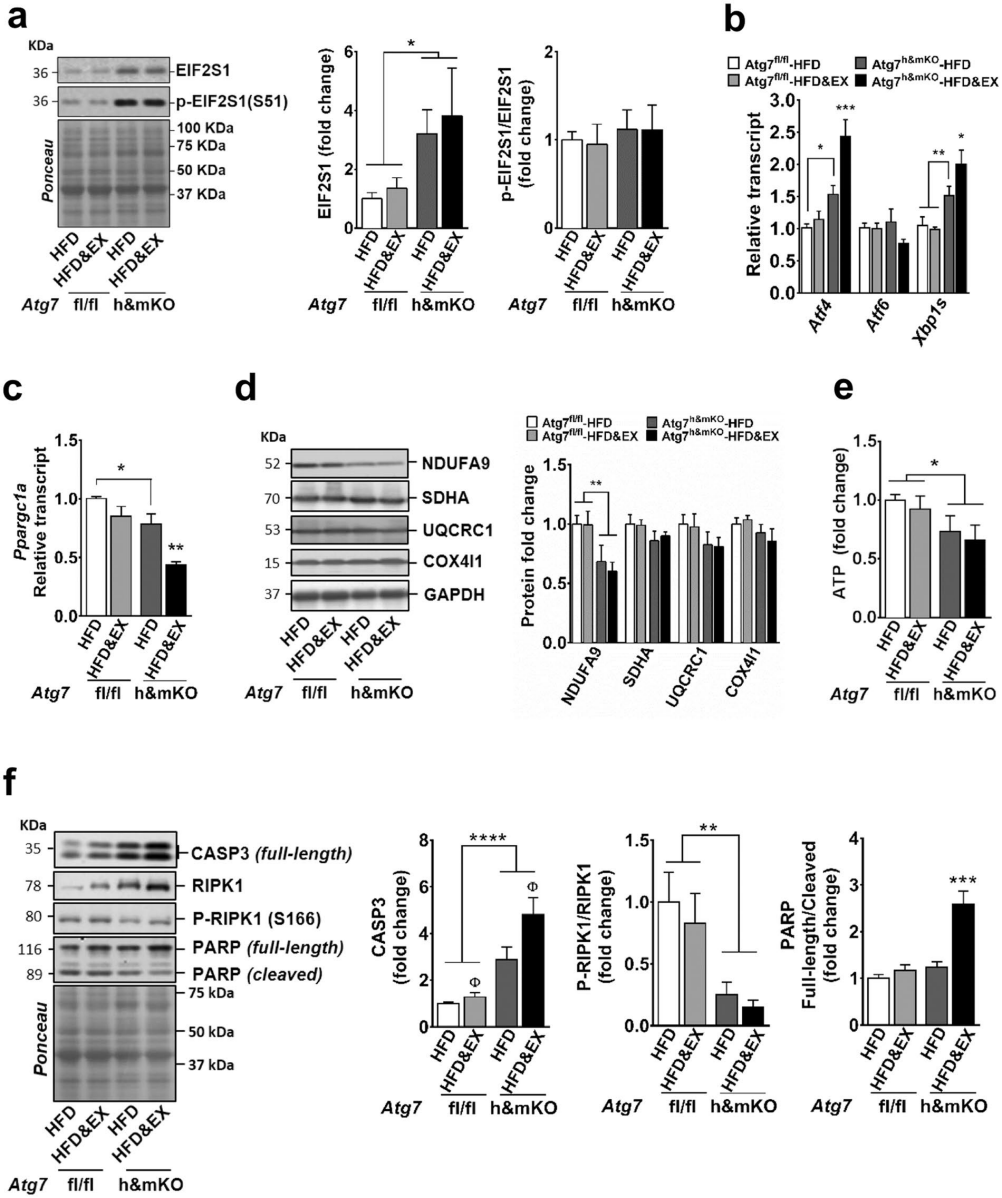
Exercise aggravates ER stress and fails to rescue mitochondrial protein homeostasis in the hearts of Atg7^h&mKO^ mice. (**a**) Immunoblot representative images of the mean value and related quantification of total EIF2S1 normalized to ponceau staining, and phosphorylated EIF2S1(S51) normalized to total EIF2S1 (n = 5–6). (**b**) mRNA analysis of ER stress effector genes (n = 5–9). (**c**) mRNA analysis of *Ppargc1a* (n = 5–7). (**d**) Immunoblot representative images of the mean value and related quantification of mitochondrial proteins of complexes 1–4 of the Electron Transport Chain normalized to GAPDH (n = 5–6). (**e**) Quantification of ATP levels in heart homogenates (n = 5–8). (**f**) Immunoblot representative images of the mean value and quantification of select cell death-related proteins (n = 5–6). Full-length blots are presented in Supplementary Fig. S7. *P < 0.05, **P < 0.01, ***P < 0.001 and ****P < 0.001 in comparison to all other groups, unless otherwise represented; ^**Φ**^ *P* < 0.05 in comparison to respective HFD only, sedentary group.

Nevertheless, we observed a trend towards elevated nitrotyrosine levels suggesting increased oxidative damage to cardiac proteins (Supplementary Fig. S3b), as well as aggravated ER stress with a substantial increase in total levels of EIF2S1 in exercise-trained Atg7^h&mKO^ mice. This was accompanied by further upregulations of both *Atf4* and *Xbp1s* (Fig. 6a,b). Interestingly, both sedentary and exercise-trained Atg7^h&mKO^ mice had reduced cardiac NDUFA9 and ATP levels suggestive of mitochondrial complex 1 protein deficiency and impaired mitochondrial function, respectively (Fig. 6d,e). Despite the increased energy demand of the myocardium with exercise, training further reduced *Ppargc1a* by ~44% in Atg7^h&mKO^ mice indicating an aggravated impairment of mitochondrial biogenesis (Fig. 6c). The hearts of exercise-trained Atg7^h&mKO^ mice also had markedly reduced levels of the PPARGC1α target *Fndc5* ^33^ and the pro-survival *Il15* ^34^ (Supplementary Fig. S3c). Finally, this unfavourable cardiac phenotype caused by exercise was not associated with either caspase 3 (CASP3)-mediated apoptosis or RIPK1-associated necroptosis^35^. This was indicated by undetectable levels of cleaved (active) CASP3 (not shown), and reduced activating phosphorylation of RIPK1 (S166), despite increased RIPK1 levels, in exercise-trained Atg7^h&mKO^ mice. However, in line with low ATP levels and defective mitochondrial protein homeostasis, the hearts of exercise-trained Atg7^h&mKO^ mice displayed a higher proportion of full-length poly (ADP-ribose) polymerase (PARP; Fig. 6f) potentially indicating a role for PARP-dependent cell death (via necrosis and/or apoptosis)^36–38^. Altogether these findings suggest that exercise training further compromised mitochondrial protein homeostasis in autophagy deficient cardiac muscle by exacerbating mitochondrial biogenesis defects, thereby elevating the metabolic stress in the hearts of Atg7^h&mKO^ mice.

## Discussion

**Figure 7:**
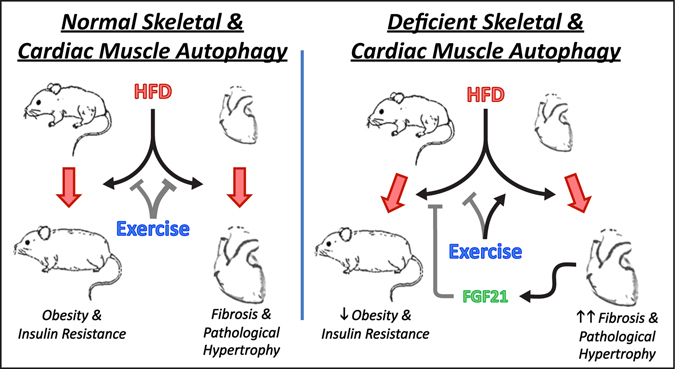
Autophagy is essential for the beneficial cardiac remodelling caused by exercise training in obesity and insulin resistance. Exercise training ameliorates diet-induced obesity and insulin resistance, and is very effective in preventing cardiac fibrosis and pathological hypertrophy when cardiac and skeletal autophagy is normal. Conversely, deficient skeletal and cardiac muscle autophagy dissociates the whole-body metabolic benefits of exercise (e.g., protection against obesity and insulin resistance) from cardioprotection. This phenomenon is driven primarily by the exercise training-mediated impairment of mitochondrial protein homeostasis, as well as exacerbation of ER stress, fibrosis and pathological hypertrophy in the hearts of mice with deficient autophagy. This unfavourable cardiac remodelling, however, potentiates the metabolic benefits of exercise in the periphery, at least in part, by elevating circulating FGF21 levels. Arrows represent stimulation, whereas T-shaped connectors represent inhibition. Thicker lines indicate stronger observed effects.

The Atg7^h&mKO^ mouse model allowed the examination of specific interactions between autophagy, exercise training, and HFD feeding in skeletal and cardiac muscles without potential confounding effects of systemic gene deletions and/or drug off-targets. In that context, two major findings arise from this investigation (Fig. 7). First, skeletal and cardiac muscles with impaired autophagy diverge in their capacity to adapt to exercise training in an obese and insulin resistant state. In fact, our results demonstrate for the first time that the role of autophagy in the heart is so instrumental for the beneficial effects of exercise, that autophagy deficiency causes exercise training to exacerbate cardiomyopathy. Second, exercise training stimulates the unfavourably remodelling heart to produce FGF21, thereby modulating the metabolic phenotype of brown adipose tissue. This heart-brown adipose tissue signalling axis increases thermogenesis and fatty acid oxidation in brown adipocytes reducing the metabolic burden on several remote tissues including visceral epididymal adipose tissue, liver, and skeletal muscle. Thus, exercise-trained Atg7^h&mKO^ mice are better protected against diet-induced obesity and insulin resistance when compared to exercise-trained control mice.

Our findings that sedentary adult Atg7^h&mKO^ mice became as obese and insulin resistant as control mice (Figs 1 and 2) contrasts with a previous study, where skeletal muscle-specific loss of *Atg7* was protective against obesity and insulin resistance^14^. The reasons for these discrepancies may be due to different efficiencies in *Atg7* gene deletion, and the consequent degree of autophagy impairment between the two studies. The Atg7^h&mKO^ mice consisted in a hypo-morphic cardiac and skeletal muscle mouse model with only partial 40% and 55% reductions of *Atg7* mRNA in these tissues, respectively. Correspondingly, *Atg7* heterozygous mice with ~50% reduction in *Atg7* mRNA in most tissues do not present a lean phenotype^39^.

Deficient autophagy compromised cellular and mitochondrial protein homeostasis in both skeletal and cardiac muscles of obese sedentary Atg7^h&mKO^, as evidenced by impaired *Ppargc1a*, aberrant mitochondrial protein expression, and ER stress; nonetheless, these tissues responded differently to exercise training. In skeletal muscle, training restored *Ppargc1a*, improved mitochondrial protein expression, and partially reversed ER stress (Fig. 4). In cardiac muscle, exercise training further reduced *Ppargc1a*, failed to rescue NDUFA9 as well as ATP levels, and exacerbated ER stress (Fig. 6). Since the metabolic demands of the myocardium are largely dependent on oxidative resynthesis of ATP, these results indicate that the maladaptive mitochondrial responses in the heart of exercise-trained Atg7^h&mKO^ mice likely played a major role in the different phenotypes observed between skeletal and cardiac muscles. The exacerbated ER stress and enhanced FGF21 expression in the myocardium of these animals provide further support to this notion as both of these can result from mitochondrial dysfunction^14, 40^. The precise mechanisms behind the maladaptive mitochondrial responses to exercise training in the hearts, but not in skeletal muscle, of Atg7^h&mKO^ are likely multifactorial. It is possible that the beneficial effects of exercise may require changes in substrate selection (e.g. enhanced glycogen breakdown)^41^ as well as increases in protein degradation that rely more heavily on autophagy in the heart versus skeletal muscle of obese, insulin resistant mice. Limitations in these processes may have substantially impacted energy consuming processes such as mitochondrial biogenesis in the hearts of Atg7^h&mKO^ mice.

While the functional implications of the unfavourable cardiac remodelling were not examined in the present study, the hearts of exercise-trained Atg7^h&mKO^ mice had several biochemical and structural features consistent with cardiomyopathy in humans and mouse models, including increased cardiac mass^42^ accompanied by mitochondrial complex 1 deficiency^43, 44^, reduced *Ppargc1a* ^45^, foetal gene reprogramming^46^, and fibrosis^47^. Perhaps related to these unfavourable cardiac changes, a subset of exercise-trained Atg7^h&mKO^ mice experienced spontaneous mortality during the intervention (data not shown). Interestingly, spontaneous early death was previously observed in mice with adipose-specific, and skeletal muscle-specific deletion of *Atg7* ^48, 49^. Although the cause of death remains unknown in each of these mouse models, altogether these observations underscore the importance of autophagy as a multi-organ survival process under conditions of metabolic stress. Future studies examining the molecular regulation of cardiac autophagy by exercise, as well as the cellular pathways directly impacted in this context, should reveal novel therapeutic targets for cardioprotection in obesity and type 2 diabetes.

Notably, exercise-trained Atg7^h&mKO^ had elevated circulating FGF21 in both fasted and randomly fed states. Although other unknown factors might have contributed to the improved metabolic phenotype of Atg7^h&mKO^ with exercise training, the BAT and eWAT metabolic gene profiles of these mice were consistent with the ones observed in conditions of HFD feeding in FGF21 transgenic mice, where increased *Ucp1* was documented in BAT and subcutaneous white adipose tissue (scWAT), but not in eWAT^50^. Therefore, an untested role for the scWAT in the metabolically superior phenotype of exercise-trained Atg7^h&mKO^ mice is also plausible. Under normal physiological conditions circulating FGF21 is primarily derived from the liver^51^. However, FGF21 can also be produced by adipose tissues^52^, skeletal muscle^14^, and heart^53^ under conditions of metabolic burden and/or mitochondrial dysfunction that lead to ER stress. Mechanistically, elevated ATF4 resulting from increased ER stress stimulates *Fgf21* in skeletal muscle^14^. ER stress has also been shown to stimulate *Fgf21* in the heart^54^. Here, we observed a marked increase in cardiac *Atf4* in exercise-trained Atg7^h&mKO^ mice, further suggesting a potential link between cardiac ATF4 and FGF21. Nevertheless, studies on the molecular regulation of cardiac *Fgf21* in conditions of metabolic stress are still required.

In conclusion, our findings provide evidence for a complex relationship between autophagy, tissue function, and whole-body metabolic homeostasis. Using a preclinical model of obesity and pre-diabetes, our results reveal that normal levels of cardiac autophagy are absolutely required for the metabolic benefits of exercise training in the heart. Exercise training causes the heart with impaired autophagy to undergo pathological hypertrophy and marked fibrosis that occur in parallel with aberrant mitochondrial protein expression, reduced ATP availability, and exacerbated ER stress. Consequently, cardiac muscle produces FGF21, which stimulates thermogenesis and lipid oxidation in brown adipose tissue improving whole-body metabolic homeostasis. Of note, our findings provide evidence for enhanced whole-body metabolic homeostasis when exercise training is performed with concomitant elevation of circulating FGF21 levels. Therefore, the potential combined use of exercise and FGF21 as therapy for chronic metabolic diseases such as obesity, diabetes, and the metabolic syndrome deserves further examination.

## Materials and Methods

### Animal models

Mice homozygous for the *Atg7* LoxP flanked allele (Atg7^fl/fl^) on a C57BL6 background were previously described^12^ and heart (cardiac)- and muscle-specific Atg7 knockout (Atg7^h&mKO^) mice were obtained by crossing the Atg7^fl/fl^ with C57BL6 mice expressing Cre recombinase driven by the muscle creatine kinase promoter (*Ckm-*Cre) from The Jackson Laboratory (Stock # 006475). Male mice (Atg7^h&mKO^[Atg7^fl/fl^; *Ckm*-Cre^tg/0^] and littermates Atg7^fl/fl^ [Atg7^fl/fl^; *Ckm*-Cre^0/0^]), 12–14 weeks of age were used in the study. Mice were housed in temperature-controlled (21 °C) quarters with a 12:12 h light-dark cycle and free access to water and standard chow (Harlan 7912; Harlan Bioproducts) unless otherwise indicated. Tissue samples were harvested after mice were anesthetized with isoflurane followed by euthanasia with cervical dislocation. All animal protocols were approved by the Institutional Animal Care and Use Committees from the University of Virginia and the University of Iowa, and experiments were performed in accordance with the guidelines for the Care and Use of Laboratory Animals of these institutions.

### Exercise training and high-fat diet (HFD) feeding protocol

Atg7^fl/fl^ and Atg7^h&mKO^ were randomly divided into groups with HFD feeding (60% calories from fat, Research Diets, New Brunswick, NJ, cat. D12492) with or without access to running wheels on their cage. Animals were acclimated to cages with wheels locked for 2 days. Wheels were unlocked on the third day, and running activity was monitored continually during the experiment (12 weeks). Wheels were then locked for 28–30 h before animals were sacrificed and tissue samples collected. Food intake was monitored daily, and feed efficiency was calculated as the ratio between body weight gain (g*1000) and total food intake during the intervention (kJ).

### Fasting glucose, ketone, insulin and calculation of the homeostasis model assessment of insulin resistance (HOMA-IR)

Fasting circulating glucose (Ascentia Contour, Bayer, Mishawaka, IN) and ketone (Nova Max, Nova Diabetes Care Inc., Billerica, MA) were assessed directly from tail vein blood after an 18 h overnight fast starting at 10 h within the light cycle (~4 pm) of the previous day. Insulin was assessed in serum obtained after blood was kept on ice for at least 30 min and then centrifuged (1,000 × g, 10 min, 4 °C), and was measured with the Ultra Sensitive Mouse Insulin ELISA Kit (Crystal Chem, Downers Grove, IL) according to the manufacturer’s instructions. HOMA-IR was calculated based on fasting glucose and insulin levels as previously described^55^.

### Glucose and insulin tolerance tests

Glucose tolerance tests (GTTs) were performed after a 6 h fasting starting 2 h into the light cycle (~8 am). Briefly, mice were transferred to clean cages without food at the beginning of the fasting period, and the test was initiated with a baseline assessment of glycaemia as described above. This was followed by an intraperitoneal injection of a glucose solution prepared in saline (2 g/kg body weight). Subsequent assessments of blood glucose levels were performed at 15, 30, 60, and 120 min after the glucose injection. Insulin tolerance tests (ITTs) were performed 8 h into the light cycle (~2 pm), and animals did not have access to food during the test. Glucose assessments were performed as described for GTTs. Briefly, the test consisted of a baseline assessment of blood glucose, followed by an intraperitoneal injection of insulin solution prepared in saline (1 U/kg of body weight; Novolin R, Novo Nordisk A/S, Bagsvaerd, Denmark). Blood glucose was assessed at 15, 30, and 60 min after the insulin injection.

### ***In vivo*** insulin signalling assessment in skeletal muscle

Mice were anesthetized via inhaled isoflurane (1.5–2%) within 3–4 h into the light cycle. Briefly, with mice under anaesthesia, muscle insulin signalling was assessed by comparing muscles of one leg (before IP injection of insulin) with muscles of the contra-lateral leg (10 minutes after i.p. injection of insulin 5 U/kg of body weight). Muscles from both legs were immediately frozen in liquid N_2_ for further analyses.

### Endurance exercise capacity test

This test was performed as previously described^11^. Essentially, mice were acclimated to treadmill running for 3 d (13.4 m/min, 10 min). On the fourth day, animals were tested with an initial speed of 13.4 m/min and 5% incline. The speed was increased by 2.7 m/min every 30 min until reaching the speed of 26.8 m/min. A brush located at the end of the treadmill was used to encourage the animals to run, and the test was terminated when a mouse stopped responding to tail brushing continuously for 20 s. Blood lactate level, used as a biochemical parameter to monitor exhaustion, was assessed before and at the termination of the test by tail bleeding using the Lactate Scout meter (SensLab, Leipzig, Germany).

### RNA extraction and quantitative real-time polymerase chain reaction (qPCR)

Total RNA was extracted from tissues with TRIzol reagent (Invitrogen), and 1 μg was reverse-transcribed using the High Capacity cDNA Reverse Transcription Kit (Applied Biosystems, Foster City, CA). qPCR was performed with a mixture containing the resulting cDNA, primers, and the Power SYBR Green PCR Master Mix in a 7500 Fast Real-time PCR System (Applied Biosystems, Foster City, CA). The primer sequences used are listed in Supplementary Table S1. The purity of each amplified product was confirmed by melting curve inspections after amplification. Results were normalized to control genes that did not change with our interventions (i.e., ribosomal protein S16 [*Rps16*, a.k.a. 16 *S*] for heart, gastrocnemius muscle, and liver; TATA-box binding protein [*Tbp*] for white adipose tissue; and actin beta [*Actb*, a.k.a. *β-actin*] for brown adipose tissue) and are presented as fold change in relation to control mice (Atg7^fl/fl^).

### Immunoblot analysis

Heart and skeletal muscle samples (plantaris and soleus muscles, unless otherwise specified) were snap-frozen in liquid nitrogen. Hearts were powdered, and an aliquot of ~15 mg was used for protein analysis; plantaris and soleus muscles were homogenized as a whole. Tissues were processed using glass homogenizers in ~17 μL/mg of ice-cold protein loading buffer containing 50 mM Tris·HCl, pH 6.8, 1% sodium dodecyl sulphate (SDS), 10% glycerol, 20 mM dithiothreitol, 127 mM 2-mercaptoethanol, and 0.01% bromophenol blue, supplemented with cOmplete Mini protease inhibitor mixture (Roche Applied Biosciences, Indianapolis, IN) and Phosphatase Inhibitor Cocktails 2 and 3 (Sigma-Aldrich, St. Louis, MO). Tissue lysates were subsequently heated for 5 min at 95 °C, centrifuged for 5 min at 15,000 rpm at room temperature to precipitate debris, and then stored in a new tube at −80 °C. Protein concentration of each sample was determined using the RC DC assay (BioRad, Hercules, CA). An equal amount of protein from each sample was subjected to SDS-polyacrylamide gel electrophoresis (SDS-PAGE) and transferred to nitrocellulose membrane. When probing for MAP1LC3, polyvinylidene difluoride (PVDF) membranes were used instead. Proteins were immunodetected using WesternSure Premium Chemiluminescent Substrate (Licor, Lincoln, NE). Bands of interest were then analysed using the Image J software (U.S. National Institutes of Health, NIH). The following antibodies and respective dilutions were used: EIF2S1 (sc-81261, 1:1000), NBR1 (sc-130380, 1:1000), PINK1 (sc-33796, 1:1000) from Santa Cruz (Santa Cruz, CA), SQSTM1 (P0067, 1:1000) from Sigma-Aldrich (St. Louis, MO), phosphorylated EIF2S1 (3597, 1:1000), AKT (9272, 1:1000), phosphorylated AKT (S473, 9271, 1:1000), MAP1LC3A/B (4108, 1:1000), UB (3936, 1:1000), BECN1 (3738, 1:1000), CASP3 (9665, 1:1000), Cleaved CASP3 (9664, 1:1000), PRKN (4211, 1:1000), RIPK1 (3493, 1:1000), phosphorylated RIPK1 (S166, 31122, 1:1000), PARP (9532, 1:1000) and GAPDH (2118, 1:2000) from Cell Signaling (Danvers, MA), and NDUFA9 (ab14713, 1:1000), SDHA (ab14715, 1:1000), UQCRC1 (ab110252, 1:1000), COX4I1 (ab33985, 1:1000), FGF21 (ab171941, 1:1000), NITROTYROSINE (ab7048, 1:1000) and TUBA1A (ab7291, 1:1000) from Abcam (Cambridge, MA), TNNI1/TNNI3 (TI-4, 1:100) from Developmental Studies Hybridoma Bank (Iowa city, IA). Results for protein expression were normalized to Ponceau stain, TUBA1A, or GAPDH.

### Masson’s trichrome staining of heart sections

Transverse myocardial sections of the left ventricle were stained by Masson’s trichrome stain for visualization of fibrotic tissue as previously described^56^. Light microscopy and image capturing was performed using a Nikon Eclipse Ti-S microscope and Nikon Digital Sight DS-Ri1 Camera (Nikon, Melville, NY, USA). For quantification of fibrotic tissue, pictures from each heart section (thickness of 10 μm) were taken systematically to ensure that the entire extent of the tissue section had been covered. A total of 20 heart sections was inspected (5 hearts/group). The micrographs were analysed using the ImageJ software (NIH). The percentage of fibrotic tissue in each micrograph was measured and averaged per heart. The average value for each heart was then utilized for statistical analysis.

### Quantification of circulating NPPA, NPPB, and FGF21

Circulating NNPA and NPPB were assessed in blood serum obtained when animals were sacrificed (4–6 h within the light cycle; random feeding condition). Starting serum volumes and dilutions were optimized for each peptide, and measures were performed in duplicates according to the manufacturer’s instructions. Essentially, NPPA was assessed in a 2-fold dilution of 25 μL of serum using the Atrial Natriuretic Peptide kit (Phoenix Pharmaceuticals, Burlingame, CA). NPPB was assessed in a 5-fold dilution of 42 μL of serum using the RayBio^®^ Mouse BNP EIA Kit (RayBiotech, Norcross, GA). Preliminary studies demonstrated equivalent sensitivity and reproducibility using 17.5 to 35 μL of serum when assessing FGF21 using the Fibroblast Growth Factor 21 Mouse/Rat ELISA (BioVendor, Asheville, NC). Therefore, FGF21 was assessed both at random feeding (6-fold dilution of 35 μL of serum) and fasting conditions (after an 18 h overnight fast; 9-fold dilution of 23 μL of serum) according to the manufacturer’s instructions.

### ATP and triglyceride quantification

ATP concentration was quantified in ~10 mg of powdered heart tissue using the ATP Determination Kit (Molecular Probes, Eugene, OR). Triglyceride concentrations were assessed in ~50 mg of liver using the Triglyceride Quantification Colorimetric/Fluorometric Kit (BioVision, Milpitas, CA). In both cases sample preparation and assays were performed according to the manufacturer’s instructions.

### Statistical analysis

Results are presented as means ± SEM, and number of samples/group are provided in figure legends. Two-tail Student’s *t* test was used to compare Atg7^fl/fl^ and Atg7^h&mKO^ mice before the diet and exercise intervention, as well as when comparing food intake, total distance run, and feed efficiency between Atg7^fl/fl^-HFD&EX and Atg7^h&mKO^-HFD&EX groups. Two-way analysis of variance (ANOVA) was used for all other analyses followed by the Newman-Keuls *post-hoc* test when applicable. Values of *P* < 0.05 were considered statistically significant.

## Electronic supplementary material

Supplementary Information
