# Supplementary material for: Exercise leads to unfavourable cardiac remodelling and enhanced metabolic homeostasis in obese mice with cardiac and skeletal muscle autophagy deficiency
Source: Sci Rep. 2017 Aug 11;7:7894. doi: 10.1038/s41598-017-08480-2 (PMC5554260; doi:10.1038/s41598-017-08480-2)
Supplement: Supplementary file 1 — Supplementary Information [file 41598_2017_8480_MOESM1_ESM.pdf]

Exercise leads to unfavourable cardiac remodelling and enhanced metabolic homeostasis in obese mice with cardiac and skeletal muscle autophagy deficiency

Zhen Yan[2], Ana Kronemberger[1], Jay Blomme[1], Jarrod A. Call[2,3], Hannah M. Caster[1], Renata O. Pereira[4], Henan Zhao[2], Vitor U. de Melo[1,5], Rhianna C. Laker[2], Mei Zhang[2], Vitor A. Lira [1,2]

[1]Department of Health & Human Physiology, Obesity Research and Educational Initiative, Fraternal Order of Eagles Diabetes Research Center, University of Iowa, Iowa City, IA, US; [2]Departments of Medicine, Pharmacology, Molecular Physiology & Biological Physics, Robert M. Berne Cardiovascular Research Center, University of Virginia School of Medicine, Charlottesville, VA, US; [3]Department of Kinesiology & Regenerative Bioscience Center, University of Georgia, Athens, GA, US; [4]Department of Internal Medicine, Carver College of Medicine, University of Iowa, Iowa City, IA, US; [5]Department of Physiology, Federal University of Sergipe, São Cristóvão, SE, Brazil.

Supplementary Information

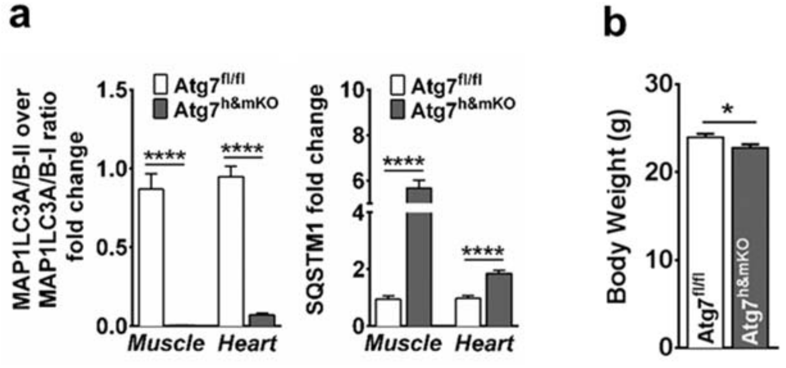

Supplementary Figure S1. Autophagy deficiency and body weight in adult Atg7$^{h\&mKO}$ mice. (**a**) Quantification of MAP1LC3A/B-II over MAP1LC3A/B-I ratio, and quantification of SQSTM1 normalized to tubulin alpha 1a (TUBA1A; n=11-15). (**b**) Body weight (n=16-17). **P* < 0.05 and *****P* < 0.0001 as represented.

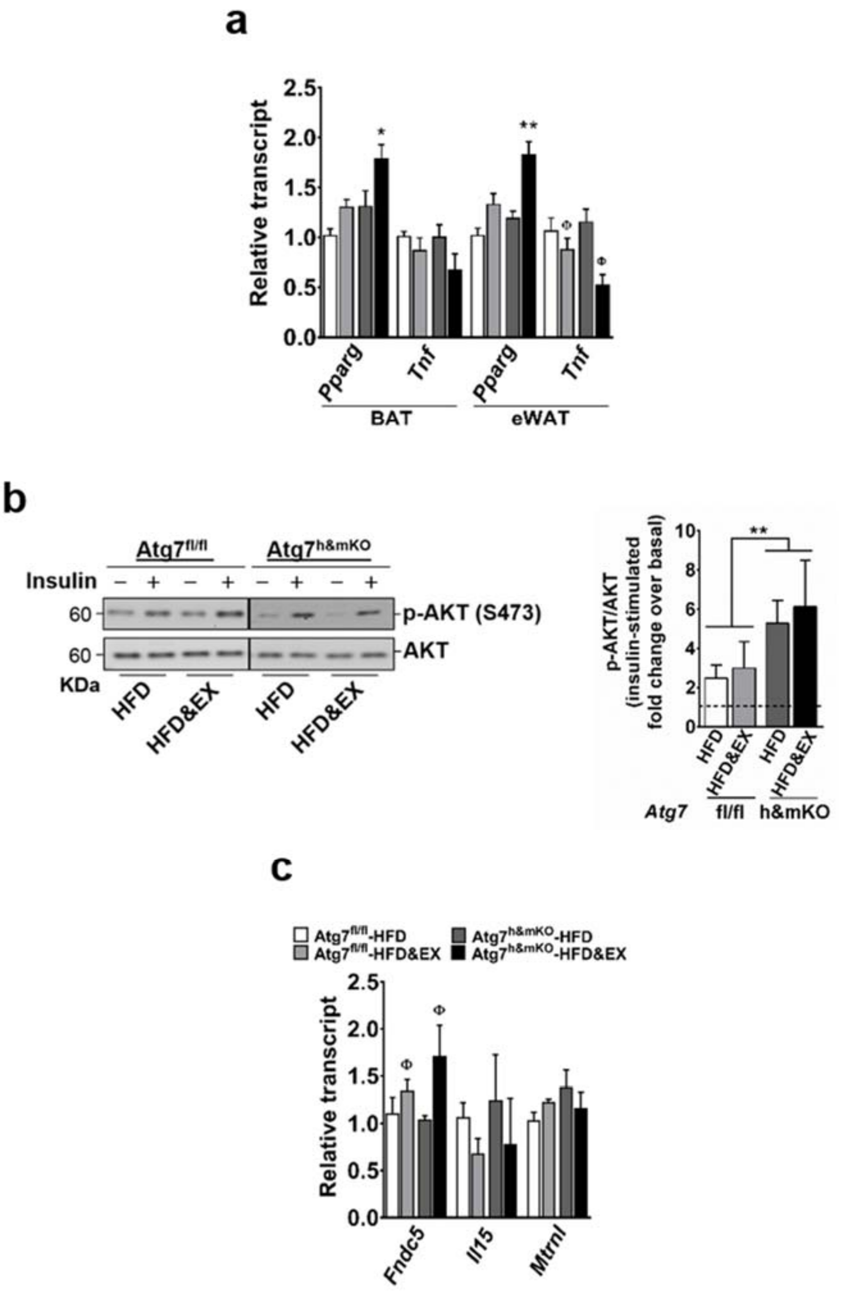

Supplementary Figure S2. Additional analyses performed in skeletal muscle, and adipose tissues of $Atg7^{fl/fl}$ and $Atg7^{h\&mKO}$ mice. (**a**) mRNA analysis of *Pparg* and *Tnf* in both BAT and eWAT (n=6-8). (**b**) Immunoblot representative images of the mean value (dividing lines indicate blots from different gels) and related quantification of phosphorylated AKT(S473) normalized to total AKT in soleus muscle under basal and insulin-stimulated conditions (dashed line represents basal AKT phosphorylation) (n=6-11). (**c**) mRNA analysis of putative myokine genes (n=5-9). $^{**}P < 0.01$ in comparison to all other groups, unless otherwise represented; and $^{\Phi}P < 0.05$ in comparison to respective HFD only, sedentary group.

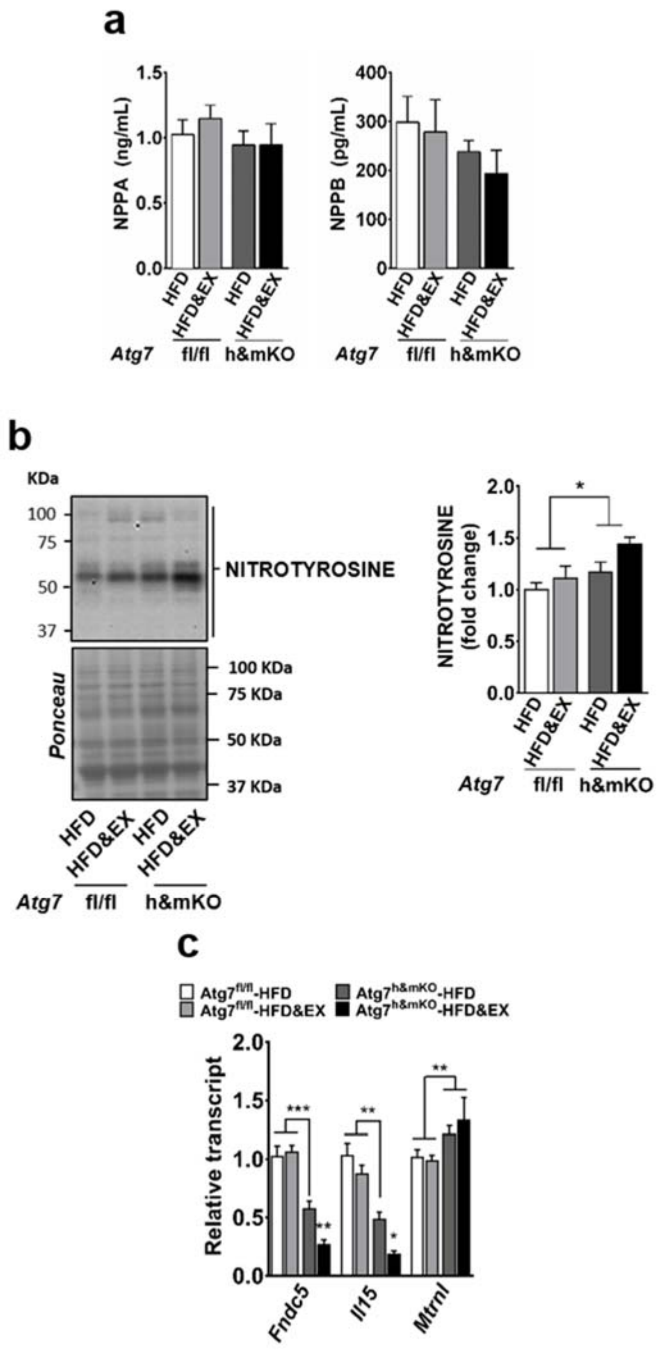

Supplementary Figure S3. Additional analyses of circulating natriuretic peptides, nitrotyrosine and myokine genes in the heart of $Atg7^{fl/fl}$ and $Atg7^{h\&mKO}$ mice. (**a**) NPPA and NPPB levels in random serum (n=5-11). (**b**) Representative cardiac nitrotyrosine blot and quantification (n=5-6). (**c**) mRNA analysis of putative myokine genes in the heart (n=5-11). *P < 0.05, **P < 0.01 and ***P < 0.01 in comparison to all other groups, unless otherwise represented.

Full-length blot of Figure 3c.

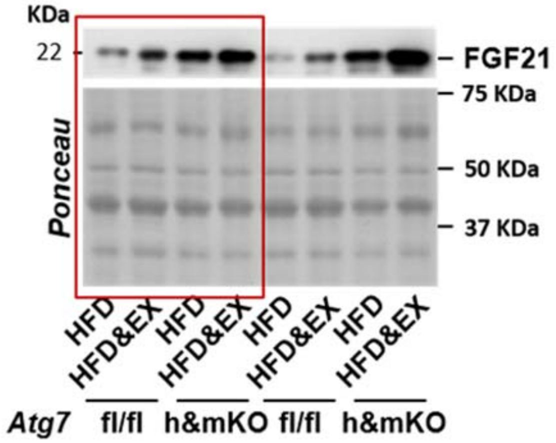

Full-length blot of Figure 3d.

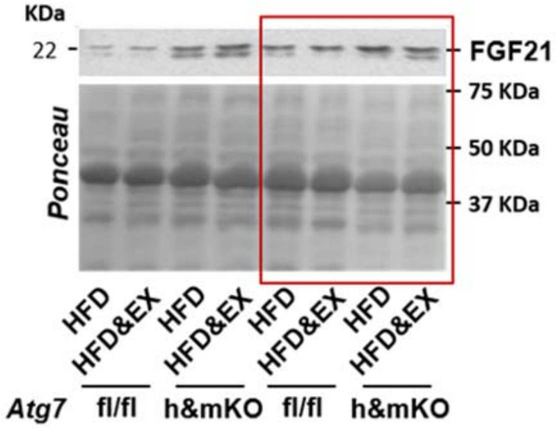

Full-length blot of Figure 3e.

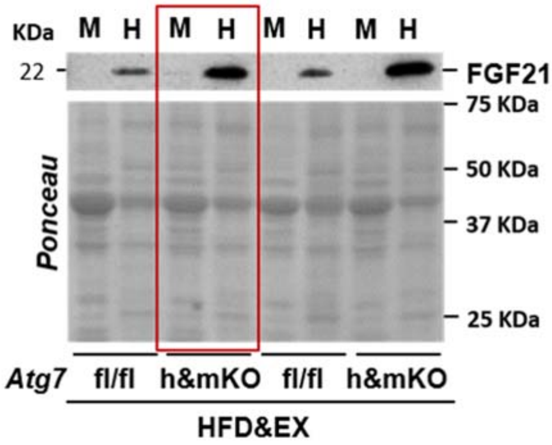

Supplementary Figure S4. Full-length blots for Figure 3.

Full-length blot of Figure 4b.

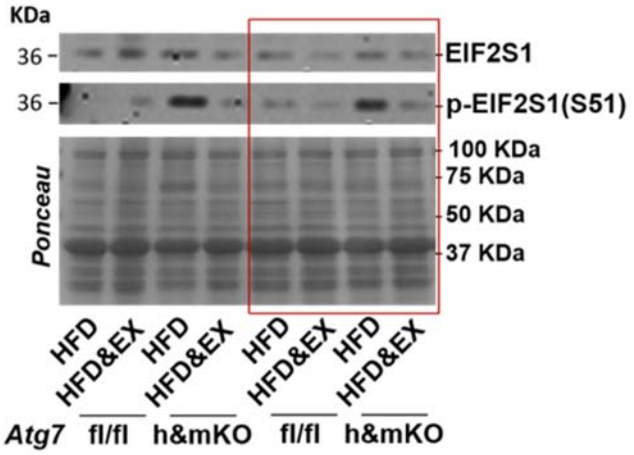

Full-length blot of Figure 4c.

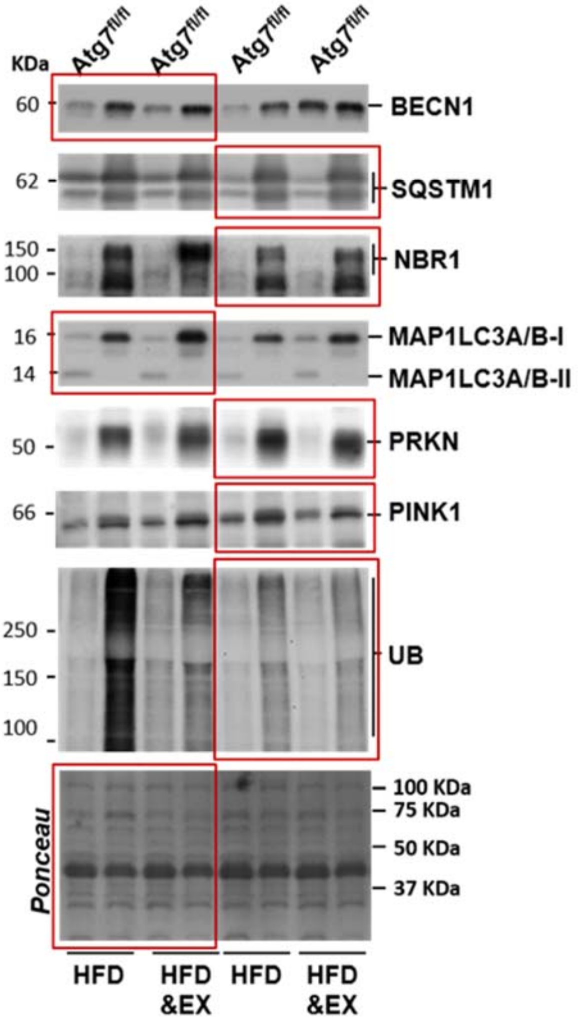

Full-length blot of Figure 4g.

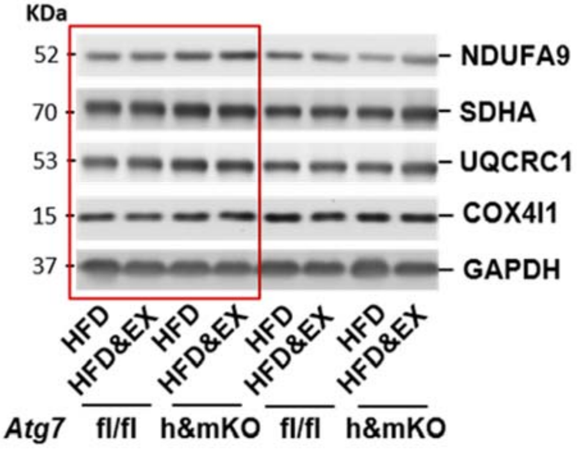

Supplementary Figure S5. Full-length blots for Figure 4.

Full-length blot of Figure 5c.

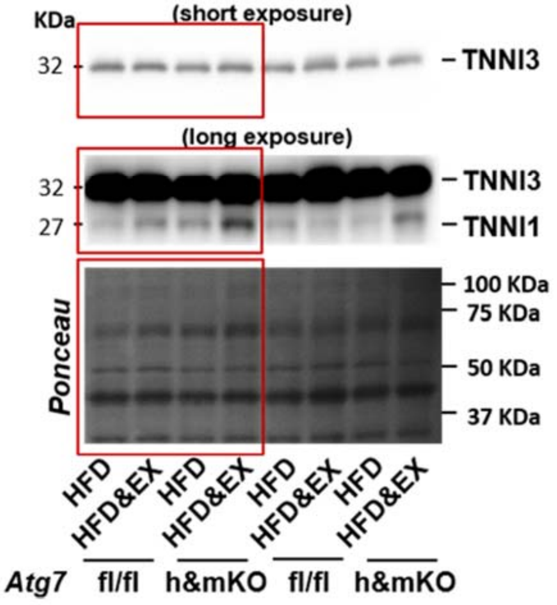

Full-length blot of Figure 5e.

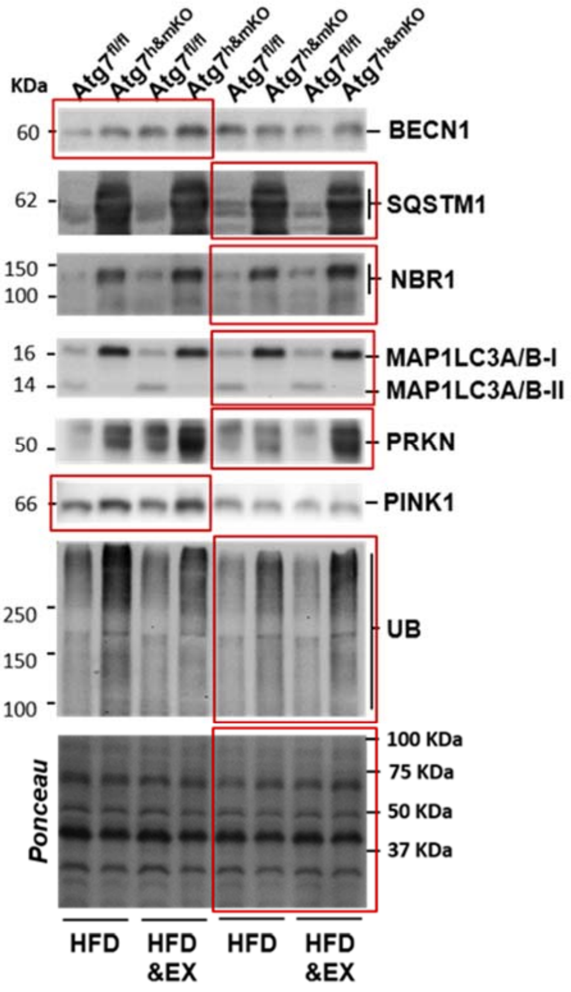

Supplementary Figure S6. Full-length blots for Figure 5.

Full-length blot of Figure 6a.

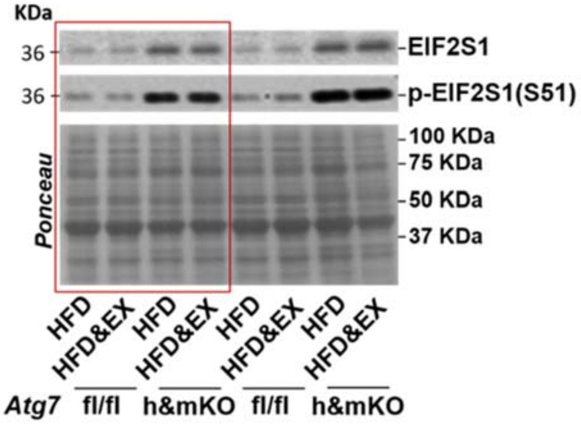

Full-length blot of Figure 6d.

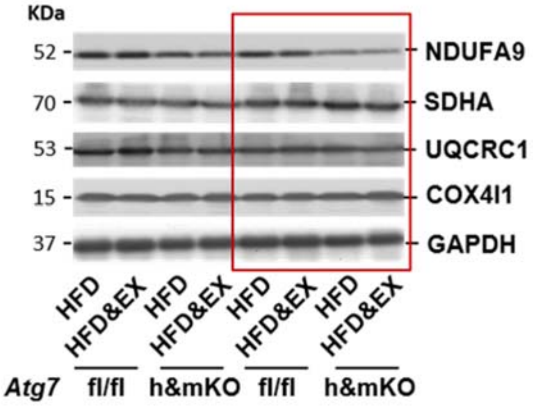

Full-length blot of Figure 6f.

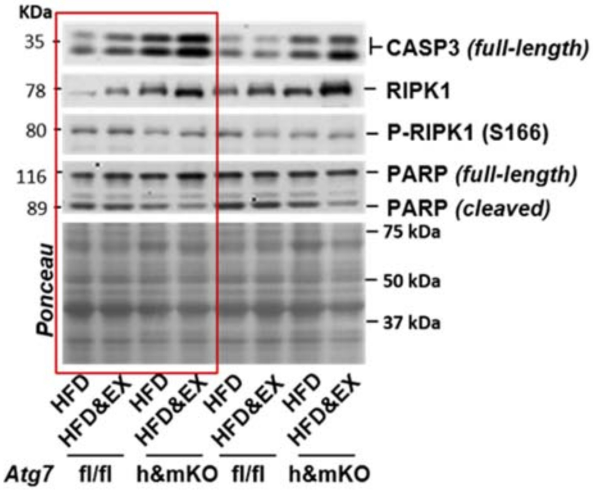

Supplementary Figure S7. Full-length blots for Figure 6.

Supplementary Table S1. Primers used in quantitative Real-time PCR.

Gene	Forward and reverse primers, 5'→3'
Acetyl-CoA carboxylase alpha (*Acaca*)	(F) TGTACAAGCAGTGTGGGCTGGCT<br>(R) CCACATGGCCTGGCTTGGAGGG
Acyl-CoA dehydrogenase, long chain (*Acadl*)	(F) GGGAATGAAAGCTCAGGACA<br>(R) AGAATCCGCATTAGCTGCAT
Acyl-CoA dehydrogenase, medium chain (*Acadm*)	(F) TGCTGGAAATGATCAACAGAAG<br>(R) CATCACCCTTCTTCTCTGCTTT
Actin, alpha1, skeletal muscle (*Acta1)*	(F) CCTGTATGCCAACAACGTCA<br>(R) CTCGTCGTACTCCTGCTTGG
Actin beta (*Actb*)	(F) AGCCATGTACGTAGCCATCCA<br>(R) TCTCCGGAGTCCATCACAATG
Activating transcription factor 4 (*Atf4*)	(F) ATGGGTTCTCCAGCGACAAG<br>(R) CCGGAAAAGGCATCCTCCTT
Activating transcription factor 6 (*Atf6*)	(F) TTCTCAGCTGATGGCTGTCC<br>(R) CTTGCAGCTCACTCCCAGAA
Adiponectin (*Adipoq*)	(F) AGACCTGGCCACTTTCTCCTCATT<br>(R) AGAGGAACAGGAGAGCTTGCAACA
Autophagy related 7 (*Atg7*)	(F) GCAAGCGAAAGCTGGTCATC<br>(R) CAGGTCAGCAGGTGCTACAA
Collagen type 1 alpha 1 chain (*Col1a1*)	(F) CATGTTCAGCTTTGTGGACCT<br>(R) GCAGCTGACTTCAGGGATGT
Connective tissue growth factor (*Ctgf*)	(F) TGACCTGGAGGAAAACATTAAGA<br>(R) AGCCTGTATGTCTTCACACTG
F-Box protein 32 (*Fbxo32*)	(F) GCAAACACTGCCACATTCTCTC<br>(R) CTTGAGGGGAAAGTGAGACG
Fibroblast growth factor 21 (*Fgf21*)	(F) TACACAGATGACGACCAAGA<br>(R) GGCTTCAGACTGGTACACAT
Fibronectin 1 (*Fn1*)	(F) CGGAGAGAGTGCCCCTACTA<br>(R) CGATATTGGTGAATCGCAGA
Fibronectin type III domain containing 5 (*Fndc5*)	(F) GAAGGAGATGGGGAGGAACCAG<br>(R) TGGCGGCAGAAGAGAGCTATAA
Interleukin 15 (*Il15*)	(F) GAATACATCCATCTCGTGCTA<br>(R) TTTGCAAAAACTCTGTGAAGG
Interleukin 6 (*Il6*)	(F) ACAAGTCGGAGGCTTAATTACACAT<br>(R) TTGCCATTGCACAACTCTTTTC
Klotho beta (*β-klotho/Klb*)	(F) TGTTCTGCTGCGAGCTGTTAC<br>(R) TTATCCCATATTGCTTTCCCGTC
Lipase E, hormone sensitive type (*Lipe*)	(F) GCTGGGCTGTCAAGCACTGT<br>(R) GTAACTGGGTAGGCTGCCAT
Meteorin like, glia cell differentiation regulator (*Metrnl*)	(F) CTGGAGCAGGGAGGCTTATTT<br>(R) GGACAACAAAGTCACTGGTACAG
Myosin, heavy chain 7, cardiac muscle, beta (*Myh7*)	(F) CGCATCAAGGAGCTCACC<br>(R) CTGCAGCCGCAGTAGGTT
Natriuretic peptide A (*Nppa*)	(F) ATGGGCTCCTTCTCCATCA<br>(R) CCTGCTTCCTCAGTCTGCTC
Natriuretic peptide B (*Nppb*)	(F) GGATCTCCTGAAGGTGCTGT<br>(R) CCTGCTTCCTCAGTCTGCTC
Patatin like phospholipase domain containing 2 (*Pnpla2*)	(F) CATGATGGTGCCCTATACTC<br>(R) GTGAGAGGTTGTTTCGTACC

Perilipin 1 (*Plin1*)	(F) GGCCTGGACGACAAAACC<br>(R) CAGGATGGGCTCCATGAC
Peroxisome proliferator activated receptor gamma (*Pparg*)	(F) ATCCCTGGTTTCATTAACCT<br>(R) GCTCCATAAAGTCACCAAAG
Peroxisome proliferator activated receptor gamma coactivator 1 alpha (*Ppargc1a*)	(F) AGCCGTGACCACTGACAACGAG<br>(R) GCTGCATGGTTCTGAGTGCTAAG
Pyruvate carboxylase (*Pc*)	(F) GGAGCTTATCCCGAACATCC<br>(R) CGGAAGACGTCCATACCATTC
Pyruvate dehydrogenase kinase 4 (*Pdk4*)	(F) CACATGCTCTTCGAACTCTTCAAG<br>(R) TGATTGTAAGGTCTTCTTTTCCCAAG
Ribosomal protein Subunit S16 (*Rps16*)	(F) TGCTGGTGTGGATATTCGGG<br>(R) CCTTGAGATGGGCTTATCGG
Solute carrier family 2 member 1 (*Slc2a1*)	(F) GCCCCCAGAAGGTTATTGA<br>(R) CGTGGTGA GTGTGGTGGAT
Solute carrier family 2 member 4 (*Slc2a4)*	(F) GTGACTGGAACACTGGTCCTA<br>(R) CCAGCCAGTTGCATTGTAG
TATA-box binding protein (*Tbp*)	(F) GAAGCTGCGGTACAATTCCAG<br>(R) CCCCTTGTACCCTTCACCAAT
Transforming growth factor beta 1 (*Tgfb1*)	(F) TGGAGCAACATGTGGAACTC<br>(R) CAGCAGCCGGTTACCAAG
Tripartite motif containing 63 (*Trim63*)	(F) TGGAAACGCTATGGAGAACC<br>(R) ATTCGCAGCCTGGAAGATG
Tumor necrosis factor alpha (*Tnfa*)	(F) ACAAGATGCTGGGACAGTGA<br>(R) TCCTTGATGGTGGTGCATGA
Uncoupling protein 1 (*Ucp1*)	(F) ACTGCCACACCTCCAGTCATT<br>(R) CTTTGCCTCACTCAGGATTGG
Uncoupling protein 2 (*Ucp2*)	(F) CTGGCAGGTAGCACCACAG<br>(R) AAAGGTGCCTCCCGAGATT
Uncoupling protein 3 (*Ucp3*)	(F) CCTCTACGACTCTGTCAAGC<br>(R) GACAGGGGAAGTTGTCAGTA
X-Box binding protein 1, spliced (*Xbp1s*)	(F) GAGTCCGCAGCAGGTG<br>(R) GTGTCAGAGTCCATGGGA
